# Evaluation of follistatin as a therapeutic in models of skeletal muscle atrophy associated with denervation and tenotomy

**DOI:** 10.1038/srep17535

**Published:** 2015-12-11

**Authors:** Patricio V. Sepulveda, Séverine Lamon, Adam Hagg, Rachel E. Thomson, Catherine E. Winbanks, Hongwei Qian, Clinton R. Bruce, Aaron P. Russell, Paul Gregorevic

**Affiliations:** 1 Baker IDI Heart and Diabetes Institute, Melbourne, 3004, Australia; 2 Centre for Physical Activity and Nutrition Research, School of Exercise and Nutrition Sciences, Deakin University, Burwood 3125, Australia; 3 Department of Physiology, Monash University, Clayton, 3800, Australia; 4 Dept. of Neurology, The University of Washington School of Medicine, Seattle 98195, USA; 5 Dept. of Biochemistry and Molecular Biology, Monash University, Clayton, 3800, Australia; 6 Dept. of Physiology, The University of Melbourne, 3010, Australia

## Abstract

Follistatin is an inhibitor of TGF-β superfamily ligands that repress skeletal muscle growth and promote muscle wasting. Accordingly, follistatin has emerged as a potential therapeutic to ameliorate the deleterious effects of muscle atrophy. However, it remains unclear whether the anabolic effects of follistatin are conserved across different modes of non-degenerative muscle wasting. In this study, the delivery of a recombinant adeno-associated viral vector expressing follistatin (rAAV:Fst) to the hind-limb musculature of mice two weeks prior to denervation or tenotomy promoted muscle hypertrophy that was sufficient to preserve muscle mass comparable to that of untreated sham-operated muscles. However, administration of rAAV:Fst to muscles at the time of denervation or tenotomy did not prevent subsequent muscle wasting. Administration of rAAV:Fst to innervated or denervated muscles increased protein synthesis, but markedly reduced protein degradation only in innervated muscles. Phosphorylation of the signalling proteins mTOR and S6RP, which are associated with protein synthesis, was increased in innervated muscles administered rAAV:Fst, but not in treated denervated muscles. These results demonstrate that the anabolic effects of follistatin are influenced by the interaction between muscle fibres and motor nerves. These findings have important implications for understanding the potential efficacy of follistatin-based therapies for non-degenerative muscle wasting.

Loss of skeletal muscle mass (atrophy) and force-producing capacity is associated with many congenital neuromuscular disorders^1^,^2^,^3^,^4^,^5^,^6^, degeneration or loss of motor nerves^7^, advanced aging^8^,^9^ and many chronic diseases^9^. Individuals with significantly reduced muscle mass and compromised muscle function commonly experience a reduced quality of life and increased mortality rate, associated with exacerbation of primary illness and increased risk of developing secondary medical complications. As many patients suffer from diseases where correction of the underlying aetiology is not yet possible, therapies aimed at preserving and/or increasing muscle mass and contractile capacity may represent viable strategies to ameliorate atrophy, and achieve positive health outcomes by reducing disease severity.

Myostatin and activin are ligands of the Transforming Growth Factor-beta (TGF-β) superfamily that repress skeletal muscle growth^10^. Myostatin ablation, *via* naturally occurring mutations or genetic knock-out, has profound anabolic effects on skeletal musculature in multiple species including mice^11^, cattle^12^, sheep^13^, and humans^14^. Myostatin and activin exert their biological effects *via* canonical Smad2/3 signalling^15^, as well as non-canonical pathways, such as modulation of signalling downstream of the serine/threonine kinase Akt^16^. Increases in Akt activity can promote muscle growth, by recruiting mammalian target of rapamycin (mTOR) signalling to stimulate protein synthesis^17^,^18^. Akt can also attenuate protein degradation^19^ by inhibiting the Fork Head family of transcription factors (FOXO1/3) that promote transcription of the muscle specific E3 ubiquitin ligases, MuRF and Atrogin-1/Mafbx^19^,^20^,^21^,^22^,^23^,^24^. Accordingly, inhibition of myostatin/activin signalling is a promising prospective approach to treating muscle wasting^25^.

Follistatin (Fst) is a naturally occurring antagonist of myostatin and activin A^26^. Overexpression of Fst promotes skeletal muscle hypertrophy in healthy rodents^11^,^27^,^28^,^29^ and primates^30^. In the setting of disease, increasing follistatin expression in musculature has proven beneficial for improving aspects of pathology in dystrophin-deficient *mdx* mice that model Duchenne and Becker muscular dystrophy (DMD, BMD)^27^. Administration of recombinant follistatin has also been shown to promote muscle hypertrophy in wild-type mice^31^, and ameliorate the progression of a mouse model of spinal muscular atrophy (SMA)^32^. We have shown that administration of recombinant adeno-associated viral vectors carrying an expression cassette for the 288aa isoform of Follistatin (rAAV:Fst) markedly increases protein synthesis, muscle mass and force-producing capacity in healthy adult mice, which is mediated in part *via* Akt/mTOR/S6K signalling^29^. These and other studies suggest that Fst has the potential to preserve/augment muscle mass and function, and provide a sound rationale for the evaluation of follistatin-based interventions as therapies for neuromuscular disorders in human trials^33^. In anticipation that positive outcomes from clinical trials would promote interest in the applicability of follistatin as a therapeutic for other conditions where muscle wasting is prevalent, it is necessary to determine to what extent Fst-based interventions can ameliorate acquired, non-degenerative muscle wasting.

Non-degenerative muscle atrophy occurs following experimental resection or inactivation of supporting motor nerves^34^. Muscle wasting induced by denervation models some (though not all) features of atrophy associated with nerve trauma^35^, sustained blockade of neuromuscular synapse activity^36^, and progressive impairment of the pre-synaptic architecture as often observed in neurodegenerative diseases, such as amyotrophic lateral sclerosis (ALS)^37^,^38^,^39^. In these settings, muscles undergo profound atrophy as a consequence of loss of electrical activity and nerve-derived trophic influences^37^,^39^. A contrasting mode of non-degenerative wasting is that based on mechanical unloading, the aspects of which can be modelled in mice by hind-limb suspension, limb casting, or tenotomy^34^. These models each differ in their recapitulation of human unloading atrophy, but all cause wasting that differs from denervation atrophy in that denervated muscles are passively loaded in the absence of nerve stimulation^34^, whereas mechanically unloaded muscles remain supported by nerve input^34^,^40^,^41^. In the study described herein, we examined whether administration of rAAV:Fst to mouse limb muscles was protective in settings of denervation- and tenotomy-induced muscle atrophy, as models of non-degenerative muscle wasting of differing aetiology. These studies demonstrate that muscles deprived of functioning motor nerves demonstrate a markedly reduced anabolic response to follistatin-based interventions intended to ameliorate atrophy.

## Results

### rAAV:Fst induces profound skeletal muscle hypertrophy *in vivo*

**Figure 1:**
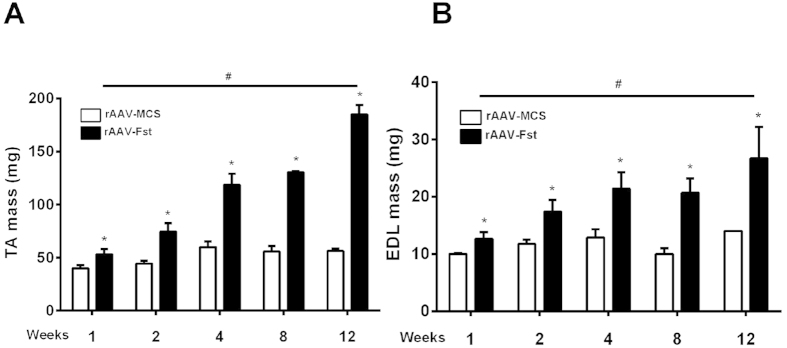
Follistatin expression induces profound skeletal muscle hypertrophy *in vivo.* The effect of rAAV:Fst on TA (**A**) and EDL (**B**) muscle mass when measured 1, 2, 4, 8 and 12 weeks after vector administration. *Significantly different from muscles receiving control vector (rAAV:MCS) at the same time point (p < 0.05); ^#^TA muscle mass in the rAAV:Fst group is significantly greater than the preceding week (p < 0.05), except for weeks 4–8 in (**B**).

When compared to muscles injected with rAAV:MCS control vector, a single injection of rAAV:Fst into the TA muscles of young-adult mice resulted in a 33%, 68%, 98%, 134% and 228% increase (all p < 0.05) in TA muscle mass when measured 1, 2, 4, 8 and 12 weeks post injection, respectively (Fig. 1A). Over the same time course, the mass of adjacent EDL muscles (which also received vector *via* the local administration protocol) increased by 26%, 48%, 73%, 100% and 91% (all p < 0.05), respectively (Fig. 1B).

### Pre-treatment of muscles with rAAV:Fst maintains mass relative to healthy muscle levels following subsequent denervation or tenotomy

**Figure 2:**
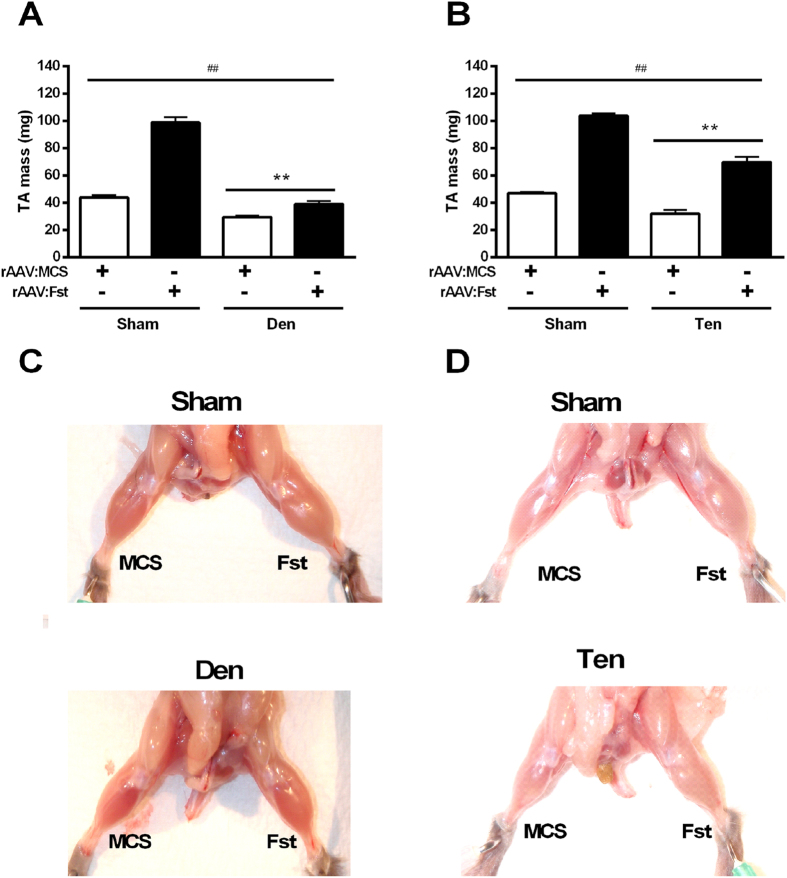
Pre-treatment of muscles with rAAV:Fst preserves mass to healthy muscle levels following denervation and tenotomy. The TA muscles of mice were administered rAAV:Fst or control vector (rAAV:MCS) 2 weeks prior to denervation (**A**) or tenotomy (**B**) surgery, and harvested for measurement of mass and other parameters 2 weeks after surgeries. **main effect for denervation or tenotomy (p < 0.01); ##main effect for rAAV:Fst treatment (p < 0.01). In addition, an interaction between group and treatment existed in (**A**) (p < 0.01) but not in (**B**). For (**A,B**), post-hoc tests revealed differences in both Sham and Den, and Sham and Ten groups, respectively (p < 0.05). Figure 2C–D shows representative images of muscles injected with rAAV:Fst or rAAV:MCS prior to denervation or tenotomy, or sham denervation/tenotomy procedures. All images were taken 2 weeks after the denervation/tenotomy/sham surgeries.

To examine the preventative effects of follistatin, TA muscles were pre-treated with rAAV:MCS or rAAV:Fst 2 weeks prior to surgical denervation. When subsequently examined 2 weeks after denervation, both denervation (Sham vs. Den) and treatment (MCS vs. Fst) had a significant effect on TA muscle mass (p < 0.01), with an additional interaction identified between group and treatment (p < 0.01). As expected, denervation of TA muscles resulted in a 32% reduction in mass when compared with innervated TA muscles. Administration of rAAV:Fst to innervated TA muscles increased mass 126% compared with innervated muscles receiving rAAV:MCS (p < 0.05). Importantly, we observed that muscles administered rAAV:Fst prior to denervation exhibited a significantly greater mass than denervated muscles receiving rAAV:MCS (p < 0.05), and were not different in mass when compared with innervated muscles previously administered rAAV:MCS (Fig. 2A).

To examine the effect of follistatin upon a model of muscle atrophy where the motor nerve is not compromised, we administered rAAV:Fst or rAAV:MCS to TA muscles prior to tenotomy (Fig. 2B). Muscles injected with rAAV:Fst 2 weeks prior to sham-tenotomy exhibited a 128% increased mass (p < 0.05) compared with muscles injected with rAAV:MCS control vector, when examined 2 weeks post tenotomy procedure. Muscles subjected to tenotomy demonstrated a 31% reduction in mass when compared to muscles undergoing sham-tenotomy (p < 0.05). Muscles administered rAAV:Fst prior to tenotomy demonstrated a marked increase in mass relative to both tenotomised and intact muscles receiving rAAV:MCS. Notably, the proportional gains in mass as a consequence of rAAV:Fst administration were not different in tenotomised vs. intact muscles. Figure 2C–D show representative images of control versus denervated or tenotomised muscles previously administered rAAV:MCS or rAAV:Fst.

### rAAV:Fst prevents atrophy in tenotomised, but not denervated muscles

**Figure 3:**
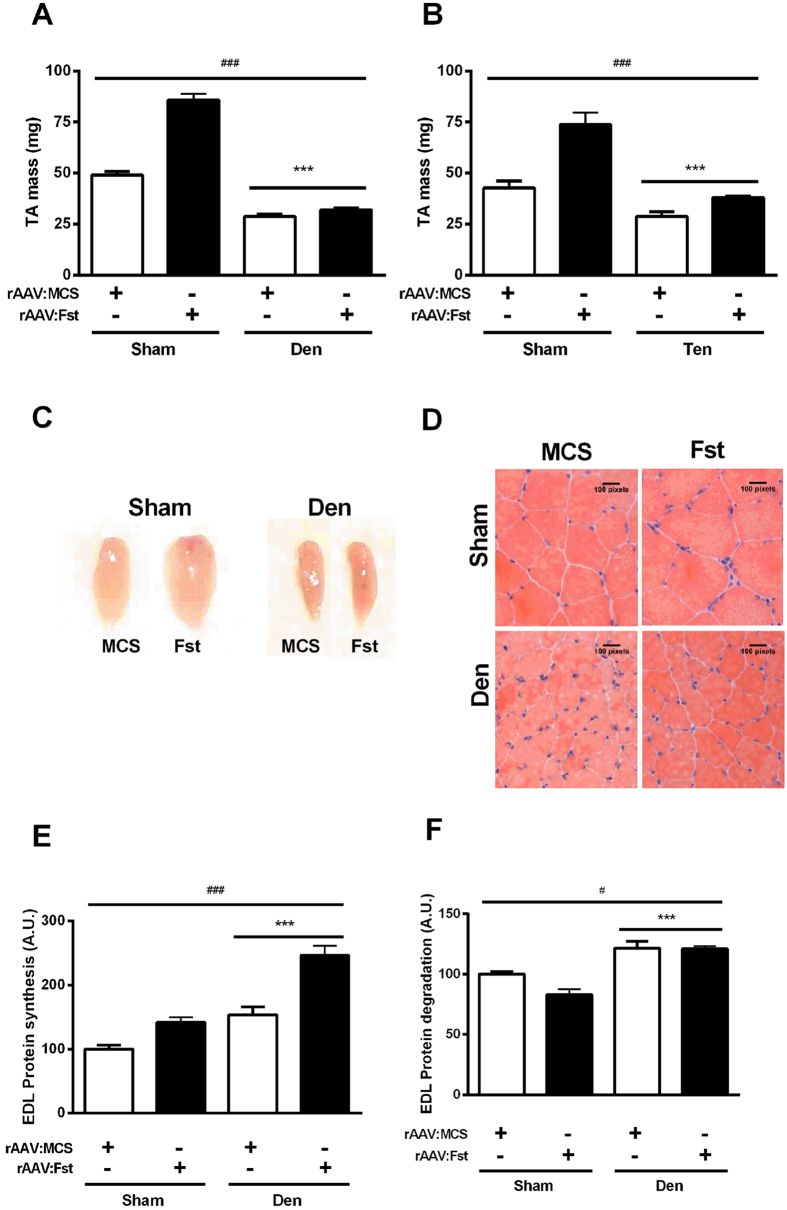
Treatment of muscles with rAAV:Fst at the time of denervation or tenotomy mitigates the atrophy of tenotomised muscles, but not denervated muscles. The TA muscles of mice were administered rAAV:Fst or control vector (rAAV:MCS) immediately prior to denervation (**A**) or tenotomy (**B**) surgery, and harvested for measurement of mass and other parameters 2 weeks after surgeries. ***Significant main effect for denervation or tenotomy on TA mass (p < 0.001); ###significant main effect for rAAV:Fst on TA mass (p < 0.001). An additional interaction between group and treatment (p < 0.01) existed in (**A**,**B**). For (**A**,**B**), post-hoc tests revealed a significant difference in both Sham and Den, and Sham and Ten groups, respectively. Representative images of whole sham and denervated TA muscles receiving AAV:Fst and AAV:MCS (**C**). Representative H&E stained sections of TA muscles examined 2 weeks after administration of rAAV:Fst and rAAV:MCS (**D**). Changes in protein synthesis (**E**) and degradation (**F**) in sham and denervated EDL muscles administered rAAV:Fst and rAAV:MCS are also shown. ***main effect for denervation on protein synthesis and degradation (p < 0.001); ### and #main effect of rAAV:Fst treatment on protein synthesis (p < 0.001) or degradation (p < 0.05). In (**E**), post-hoc tests revealed a difference in both the Sham and the Den group (p < 0.05). In (**F**), post-hoc tests revealed a difference in the Sham but not in the Den group (p < 0.05).

Having observed that hypertrophy induced by prior administration of rAAV:Fst can preserve muscle mass relative to untreated muscles, we considered the therapeutic potential of rAAV:Fst administration in a more clinically common scenario, by examining whether treatment of muscles with rAAV:Fst immediately after denervation or tenotomy could protect against subsequent atrophy. In muscles where rAAV:Fst was administered and a sham denervation was performed (i.e. nerve exposed, but kept intact), treatment increased TA muscle mass 75% by 2-weeks post-injection (p < 0.05, Fig. 3A). In contrast, muscles injected with rAAV:Fst at the time of denervation failed to prevent a ~40% reduction in TA mass (Fig. 3A). In parallel cohorts of mice studied for the interactions of rAAV:Fst and tenotomy, we observed that muscles receiving rAAV:MCS experienced a 32% reduction in mass when examined 2 weeks after treatment and tenotomy. However, muscles administered rAAV:Fst at the time of tenotomy did not differ in mass compared with intact muscles receiving control vector (Fig. 3B).

As neither changes in mass nor histology were evident in denervated muscles as a consequence of rAAV:Fst co-administration (Fig. 3C,D), we examined the effects of treatment and denervation upon subsequent protein synthesis and degradation. We found that denervation of muscles increased the rates of both protein synthesis (Fig. 3E), and protein degradation (Fig. 3F). Examining muscles for effects of rAAV:Fst administration, we found that treatment was associated with increased protein synthesis in both innervated and denervated muscles (Fig. 3E). However, rAAV:Fst administration to innervated muscles reduced protein degradation ~17% (p < 0.05), whereas treatment had no effect upon the elevated protein degradation rate in denervated muscles (Fig. 3F).

### Potentiation of mTOR signalling by Fst is impaired in denervated muscles

**Figure 4:**
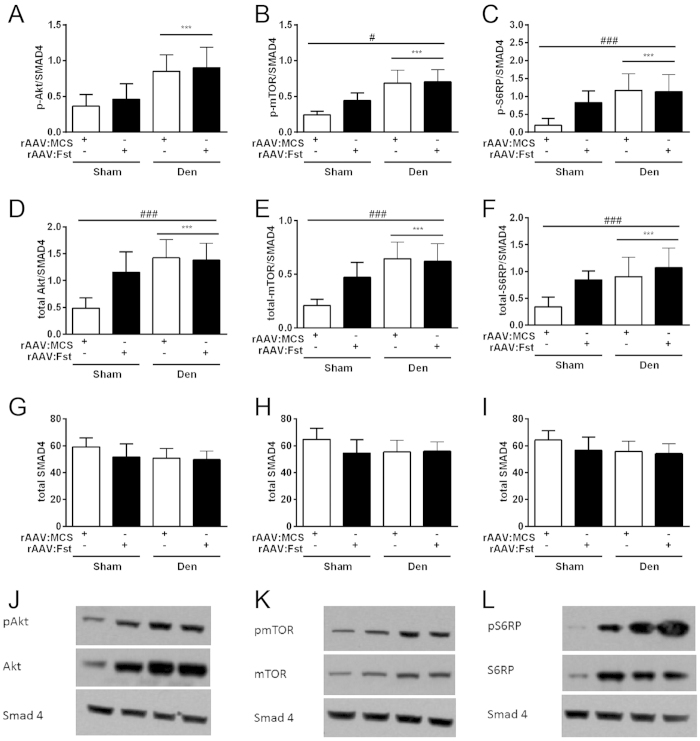
Potentiation of mTOR signalling by follistatin is impaired in denervated muscles. The effect of rAAV:Fst administration at the time of denervation on abundance of (**A**) phosphorylated Akt (p-Akt), (**B**) phosphorylated mTOR (p-mTOR), (**C**) phosphorylated S6RP (p-S6RP), (**D**) total Akt, (**E**) total mTOR and (**F**) total S6RP protein. Muscles were analysed 2 weeks after vector administration and surgery. Total SMAD4 protein levels were determined as a loading control, and are displayed in Figures 4G–4I. Representative blots presented in Figures 4J–4L. ***main effect for denervation on protein levels (p < 0.001); #and ###main effect of rAAV:Fst treatment on protein levels (p < 0.05 or p < 0.001). In (**B**), (**C**), (**D**), (**E,F**), post-hoc tests revealed a difference in the sham but not in the denervated muscles (p < 0.05).

Having established that rAAV:Fst administration to denervated muscles promoted protein synthesis, but did not attenuate protein degradation, we investigated whether treatment affected the phosphorylated and total levels of members of the Akt/mTOR signalling pathway, which are important regulators of Fst-mediated hypertrophy^29^, and of protein degradation mechanisms^17^. Consistent with our observation of increased protein synthesis occurring in denervated muscles, we detected increased abundance of phosphorylated (Fig. 4A–C) and total (Fig. 4D–F) Akt, mTOR and S6RP in denervated versus innervated muscles (all p < 0.001), but no differences in abundance of Smad4 (used here as a loading control) between cohorts (Fig. 4G–I). rAAV:Fst treatment displayed a significant main effect on the abundance of p-mTOR and p-S6RP (p < 0.05 and p < 0.001, respectively), but *post-hoc* analyses revealed significant effects of treatment in the innervated muscles only (p < 0.05 for both, Fig. 4B–C).

## Discussion

Follistatin-based interventions are presently being investigated as potential therapeutics for muscle wasting associated with a variety of neuromuscular disorders, chronic illness, and advanced aging. The data reported here provide valuable insight into the potential applicability of follistatin-based interventions as preventative or restorative therapies for muscle wasting, depending on the underlying aetiology.

Consistent with earlier studies^29^,^42^, we observed that increased expression of follistatin in mouse limb muscles stimulated muscle fibre hypertrophy. The observed effects were associated with a shift in protein turnover favouring increased anabolism. As follistatin-based interventions have proven beneficial in models of the neuromuscular disorders SMA^43^ and DMD/BMD^42^, we sought to investigate whether expression of follistatin could also protect against acquired non-degenerative muscle wasting. Studying follistatin effects in the context of muscle atrophy caused by motor nerve resection or tenotomy provided the means to evaluate the impact of different external stimuli upon follistatin-induced muscle adaptation. The experimental denervation procedure models muscle atrophy associated with disruption of the nerve-muscle interaction, while preserving the exposure of muscles to changes in length and passive loading with limb movement. Conversely, the tenotomy protocol provokes muscle atrophy associated with prolonged unloading, but maintains a functional interface between muscle fibres and motor nerves. Future studies may benefit from considering the effects of follistatin in other mouse models of unloading atrophy that maintain intact tendons, such as limb casting and hind-limb suspension.

Administration of rAAV:Fst to muscles two weeks prior to denervation or tenotomy stimulated muscle hypertrophy. Analysis of muscles after subsequent denervation or tenotomy demonstrated that muscle hypertrophy associated with pre-emptive rAAV:Fst treatment ensured that the mass of muscles remained comparable to (or exceeded) that of un-treated sham-operated muscles. These results are important because they support the potential use of follistatin-based interventions as a preventative measure to improve rehabilitation outcomes following anticipated events that may cause muscle atrophy. One such clinical example would be planned orthopaedic procedures and the ensuing recovery period in which physical activity is initially limited.

Seeking next to evaluate follistatin as a potential intervention for treating wasting following acute injury, we found that administration of rAAV:Fst to muscles at the time of tenotomy largely prevented loss of mass relative to healthy muscles. However, administration of rAAV:Fst to acutely denervated muscles achieved very limited protection against neurogenic atrophy. Mechanistically, we observed that increasing follistatin expression in innervated muscles promoted protein synthesis and attenuated protein degradation, whereas treatment of denervated muscles promoted protein synthesis, but did not diminish protein degradation. Consistent with these findings, we observed a positive effect of rAAV:Fst administration upon the key regulators of protein turnover mTOR and S6RP in innervated muscles, but not in denervated muscles. Regarding the relationship between protein synthesis and signalling via Akt/mTOR, discordance between the amplitude of signalling protein phosphorylation and biological endpoints such as muscle protein synthesis has been described previously^44^. Also, it is important to highlight that others have demonstrated muscle hypertrophy can occur *via* mTOR-independent mechanisms^45^, and that we have reported that follistatin-mediated hypertrophy of innervated muscles takes place *via* mechanisms that are not contingent upon mTOR activation^29^. It is also notable that denervation of muscles elicits marked changes in abundance and activation of the Akt/mTOR/S6K pathway, but that contention exists as to whether mTOR activity is beneficial, or exacerbates atrophy *via* negative feedback inhibition of Akt^46^,^47^,^48^. Collectively, these findings demonstrate that the mechanisms by which follistatin-based interventions exert anabolic and anti-catabolic effects upon adult skeletal muscle are influenced by the many signalling changes that occur in muscles when the interface between muscle fibres and motor nerves is compromised.

The primary mode of action by which follistatin exerts effects upon skeletal muscle has been ascribed to its targeted binding with, and inhibition of members of the TGF-β protein superfamily that negatively regulate muscle mass *via* the type II activin receptor (ActRII) and Smad2/3 signalling proteins, such as myostatin and activins A and B^29^,^31^,^49^. Expression of myostatin, TGF-β1 and -β2 has been demonstrated to increase in rodent muscles during neurogenic atrophy^50^,^51^,^52^,^53^, as has activation of the downstream Smad signalling proteins^47^, although it is less clear which other members of the TGF-β superfamily may also be regulated in this setting. Soluble recombinant ActRIIB receptors have been trialled previously as inhibitors of these ligands, and demonstrated potential as therapeutics for muscle wasting associated with some modes of muscle atrophy, such as cancer cachexia^54^. However, it is interesting to note that administration of soluble ActRIIB as an intervention for neurogenic atrophy has proven largely ineffectual to date^47^. The consistency between the outcomes of those studies using soluble ActRIIB and the results described herein point to the potent anabolic effects of inhibiting myostatin and activin actions upon skeletal muscles being heavily influenced by cellular signalling that is modulated by motor nerve activity.

The findings reported here are clinically relevant as they provide new insight into disease contexts where Follistatin-based interventions may offer benefit, or conversely have relatively little effect. For instance, conditions of muscle wasting associated with progressive degeneration of motor nerves may benefit from administration of Follistatin-based therapies in advance of significant nerve loss (where accumulation of added muscle mass and strength could bolster patients’ functional capacity to delay eventual functional declines). Conversely, attempts to restore mass and strength subsequent to significant denervation may be comparatively ineffectual. Such effects have been observed to an extent when evaluating Fst-based interventions in a mouse model of amyotrophic lateral sclerosis (ALS), or motor neuron disease^55^. Potentially, administration of Fst-based therapies to elderly citizens exhibiting severe sarcopenia may demonstrate a reduced enhancement of muscle mass and strength compared to treatment of young-adults, owing in part to a progressive deterioration of the neuromuscular junction in aging muscles^56^. However, as the incidence of denervated muscle fibres in aged muscles is greatly reduced compared with the extreme denervation observed in end-stage (ALS), it is envisaged that follistatin-based interventions may still prove beneficial for enhancing muscle mass and augmenting muscle function in the face of advanced sarcopenia. As other research suggests that administering follistatin-based interventions to muscles in advance of sarcopenia is also highly effective at preventing muscle atrophy associated with advanced aging^29^, further exploration of follistatin as a therapeutic for frailty associated with non-degenerative muscle wasting such as that which commonly impacts on the morbidity and mortality of aged citizens is supported.

In summary, the findings presented here show that follistatin is an important modulator of muscle mass than can alter processes of protein synthesis and degradation in favour of anabolism, but that these effects are compromised by disruption of the interaction between muscle fibres and their associated motor nerves. Follistatin-based interventions may offer the potential to ameliorate or compensate for non-degenerative muscle wasting often associated with chronic illness or advanced aging, but our results demonstrate that reduced interaction between muscle fibres and motor nerves as a factor in the onset and progression of muscle wasting should be considered when defining indications for the use of follistatin-based interventions. These findings advocate for further investigation into how follistatin-mediated effects upon skeletal muscles are influenced by cellular processes that are responsive to motor nerve input. Additionally, it will be important to investigate whether follistatin-based interventions could be combined with other approaches that circumvent the signalling effects of denervation, to develop superior therapies for muscle atrophy.

## Methods

### rAAV vector production

AAV expression cassette plasmids comprising rAAV2 Inverted Terminal Repeats (ITRs) flanking a) the non-specific cytomegalovirus (CMV) promoter/enhancer; b) a sequence encoding the short non-circulating form of Follistatin (Follistatin-288; pAAV:Fst) or a multiple cloning site motif (pAAV:MCS, used as a control); c) the SV40 poly-A sequence were assembled using standard cloning techniques, as described previously^29^. The resultant plasmids were used to prepare recombinant AAV vectors as reported previously^57^,^58^. Briefly, HEK293 cells were plated at a density of 3.2−3.8 × 10^6^ cells on a 10 cm culture dish, 8–16 h prior to transfection with 10 μg of a vector-genome-containing plasmid and 20 μg of the packaging/helper plasmid pDGM6, by means of the calcium phosphate precipitate method to generate pseudotype 6 vectors. Seventy-two hours after transfection, the media and cells were collected and homogenised through a microfluidiser (Microfluidics) prior to 0.22 μm clarification (Millipore). The vector was purified from the clarified lysate by affinity chromatography over a heparin affinity column (HiTrap, Amersham), and ultracentrifuged overnight prior to re-suspension in sterile physiological Ringer’s solution. The purified vector preparations were titred with a customised sequence-specific quantitative PCR-based reaction (Applied Biosystems Inc.).

### Animal handling and interventions

All experimental procedures were conducted in accordance with the relevant codes of practice for the care and use of animals for scientific purposes (National Institutes of Health, 1985, and the National Health & Medical Council of Australia, 2004) as approved by Alfred Medical Research and Education precinct Animal Experimentation Ethics Committee. Young adult (6–8 week old) male C57BL/6J mice were housed with littermates and maintained at 22 °C under 12-h light/12-h dark cycles with *ad libitum* access to chow diet and water. All surgical procedures were performed under general anaesthesia (4% isoflurane in O_2_ with 0.5% adjustment to maintain depth of anaesthesia), supported by post-surgical analgesia (Carprofen, 5 mg/kg/day).

### Denervation and intramuscular injection of vectors

After mice were deeply anesthetized (isoflurane inhaled in medical oxygen), a small skin incision was performed on the lateral surface of the lower hind limb to resect ~2 mm of the peroneal (deep fibular) nerve branch, thus denervating the *Tibialis Anterior* (TA) and *Extensor Digitorum Longus* (EDL) muscles, or to perform a sham denervation (i.e. nerve exposed but kept intact and the overlying incision re-sealed). Care was taken not to damage the adjacent blood supply, and the overlying incision was sealed with topical administration of surgical adhesive. Following denervation, the TA and EDL musculature received an injection of rAAV:Fst (or rAAV:MCS control vector in the contralateral leg) in 30 μl of Hanks Buffered Saline Solution (HBSS) which was delivered *via* longitudinal injection of the musculature occupying the anterior compartment of the hind limb through a small skin incision created overlying the distal portion of the TA and EDL muscles^29^. At the completion of vector injection, the skin surrounding the injection site was sealed with surgical adhesive (Vetbond, 3 M), and the mouse monitored for recovery of full consciousness, before being returned to its home cage.

### Tenotomy and intramuscular injection of vectors

In anesthetized mice, a small skin incision was made over the distal portion of the TA and EDL muscles to facilitate intramuscular administration of rAAV vectors. Upon completion of vector administration and closure of the skin incision, a second skin incision was performed across the dorsal surface of the hind paw to surgically expose the distal tendons of the TA and EDL muscles adjacent to the retinaculum. A ~2 mm portion of the distal TA and EDL tendons was surgically resected, and the cut ends cauterized. Upon confirmation that the TA and EDL muscles were tenotomised, the skin incision was sealed, and the animals returned to full consciousness, prior to return to their home cage. For sham tenotomy, the tendon was surgically exposed but kept intact, and the overlying incision re-sealed.

### Tissue collection

At the designated time of tissue harvest, mice were humanely euthanized via cervical dislocation to facilitate the rapid dissection of the hind limb muscles. Excised muscles were trimmed of any adherent non-muscle tissues, quickly blotted dry, and weighed to record mass. For histology, muscles were embedded in cryoprotectant (Tissue-Tek OCT medium, Sakura Finetek) and snap frozen in liquid nitrogen-cooled isopentane. For protein signalling, muscles were snap frozen in liquid nitrogen and stored at −80 °C.

### Histological examination of muscles

For histological examination of muscle morphology, frozen muscle samples were cryosectioned at 10 μm thickness and stained with haematoxylin and eosin as described previously^29^. Sections were mounted (DePeX mounting medium, BDH) and images of stained sections were captured at room temperature using a U-TV1X-2 camera mounted to an IX71 microscope, and a PlanC 10X/0.25 objective lens (Olympus). DP2-BSW acquisition software (Olympus) was used to acquire images.

### *Ex-vivo* protein synthesis assay

EDL muscles were carefully dissected with tendons intact and pre-incubated for 30 min in 2 mL of warmed (30 °C) modified Kreb’s-Henseleit buffer (KHB = 4.5% NaCl, 5.75% KCl, 6.1% CaCl_2_, 10.55% KH_2_PO4, 19.1% MgSO_4_.7H2O, 16% v/v NaHCO_3_) gassed with 95% O_2_:5% CO_2_ as described previously^59^. The intracellular protein pool was labelled as described by others^60^. Briefly, EDL muscles were transferred to a vial containing 2 mL of pulse (radioactive) buffer comprising 5 μCi/mL of ^3^H-Tyrosine (Amersham Life Sciences) and 500 mM L-Tyrosine in KHB for 1 hr. After this labelling phase, the muscles were rinsed in non-radioactive KHB, dry blotted, weighed and snap frozen in liquid nitrogen before their storage at −80 °C. Muscles were subsequently homogenized in 500 μL of 10% Trichloroacetic acid and centrifuged at 10,000 × g for 15 min at 4 °C. The pellets corresponding to the insoluble protein fractions were suspended in 500 μL of 1 M NaOH and allowed to dissolve overnight at room temperature. To measure incorporation of the radiolabel into protein, 100 μL of each sample was added into 5 mL of scintillation fluid, and ^3^H radioactivity was measured in triplicates in a scintillation counter (Beckman).

### *Ex-vivo* protein degradation assay

Protein degradation was measured using a modified version of a previously described protocol^61^. The rate of protein degradation in EDL muscles was estimated by labelling the intracellular protein pool with ^3^H-Tyrosine as described above for the protein synthesis assay. After the labelling phase, muscles were transferred to a fresh vial containing 2 mL of KHB containing protein synthesis inhibitor buffer (cold buffer supplemented with 500 mM L-Tyrosine and 30 μM cycloheximide). After 30 min (sufficient time to inhibit protein synthesis and tracer re-incorporation), the muscles were transferred to a new vial containing 2.2 mL of protein synthesis buffer. Muscle samples were incubated in this buffer for a total of 1 h. During this period, 100 μL aliquots of the incubation buffer were taken at intervals and counted in 5 mL of scintillation media. When comparing time points, measurements were adjusted for volume.

### Western Blotting

As previously described^29^, frozen muscles were homogenized with NP-40-supplemented lysis buffer (Sigma-Aldrich) supplemented with protease and phosphatase inhibitor cocktails (Sigma-Aldrich). Lysis was followed by centrifugation at 15,000 × g for 20 min at 4 °C. Protein concentration was determined using a micro protein assay kit (Pierce) according to the manufacturer’s indications. Protein samples were then mixed with 4× Laemmli’s loading buffer (4% sodium dodecyl sulphate (SDS), 20% glycerol, 10% 2-mercaptoethanol, 0.004% Bromophenol blue and 0.125 M Tris-HCl pH 8.0) and denatured for 5 min at 95 °C. Protein fractions were subsequently separated by SDS-PAGE using pre-cast 4–12% Bis-Tris gels (Invitrogen). Gels were transferred onto nitrocellulose membranes (BioRad) and blocked for 1 hour in 5% (w/v) skim milk powder in Phosphate-buffered saline (PBS) containing 0.1% Tween-20 (PBST) at room temperature. Membranes were incubated with the appropriate antibody overnight (4 °C), which was followed by several washes with PBS before using a secondary antibody for 1 hour at room temperature. Antibody-bound proteins were revealed using ECL (GE Healthcare) for 1 min and visualized with film^21^. Quantification of labelled Western blots was performed using ImageJ analysis software^62^. Densitometric analyses of Western blots are presented as band density normalised to the loading control. All antibodies used were obtained from Cell Signaling Technology.

### Statistics

All data are reported as mean ± SEM. The mixed-model 2-way analysis of variance (ANOVA) was used to compare group means using GenStat v16 VSN International. Diagnostic plots of residuals and fitted values were checked to ensure homogeneity of variance (a key assumption for ANOVA). Consequently, all data was log10-transformed and analyses were conducted on these transformed scales. The least significant difference (LSD) test was used to compare pairs of means. The significance levels for both the F-tests in the ANOVA and the LSD tests were set at p < 0.05. Note that in the figures, the reported statistical significance is based on analysis of the transformed data but the reported means ± S.E.M. are on the original (untransformed) scale.

## Additional Information

**How to cite this article**: Sepulveda, P. V. *et al.* Evaluation of follistatin as a therapeutic in models of skeletal muscle atrophy associated with denervation and tenotomy. *Sci. Rep.* **5**, 17535; doi: 10.1038/srep17535 (2015).

## References

[b1] Mokri B. & Engel A. G. Duchenne dystrophy: electron microscopic findings pointing to a basic or early abnormality in the plasma membrane of the muscle fiber. Neurology 25, 1111 (1975). 1105232 10.1212/wnl.25.12.1111

[b2] Bione S., Maestrini E., Rivella S., Mancini M. & Regis S. Identification of a novel X-linked gene responsible for Emery-Dreifuss muscular dystrophy. Nat. Genet. 8, 323 (1994). 7894480 10.1038/ng1294-323

[b3] Helbling-Leclerc A., Zhang X., Topaloglu H., Cruaud C. & Tesson F. Mutations in the laminin alpha 2-chain gene (LAMA2) cause merosin-deficient congenital muscular dystrophy. Nat. Genet. 11, 216–218 (1995). 7550355 10.1038/ng1095-216

[b4] Nigro V., de Sa Moreira E., Piluso G., Vainzof M. & Belsito A. Autosomal recessive limb-girdle muscular dystrophy, LGMD2F, is caused by a mutation in the delta-sarcoglycan gene. Nat. Genet. 14, 195–198 (1996). 8841194 10.1038/ng1096-195

[b5] Fisher J. & Upadhyaya M. Molecular genetics of facioscapulohumeral muscular dystrophy (FSHD). Neuromuscul. Disord. 7, 55–62 (1997). 9132141 10.1016/s0960-8966(96)00400-2

[b6] Ueda H., Ohno S. & Kobayashi T. Myotonic dystrophy and myotonic dystrophy protein kinase. Prog. Histochem. Cytochem. 35, 187– 251 (2000). 11064921 10.1016/s0079-6336(00)80002-9

[b7] Midrio M. The denervated muscle: facts and hypotheses. A historical review. Eur. J. Appl. Physiol. 98, 1–21 (2006). 16896733 10.1007/s00421-006-0256-z

[b8] Haddad F. & Adams G. R. Aging-sensitive cellular and molecular mechanisms associated with skeletal muscle hypertrophy. J. Appl. Physiol. 100, 1188–1203 (2006). 16373446 10.1152/japplphysiol.01227.2005

[b9] Argiles J. M., Busquets S., Felipe A. & Lopez-Soriano F. J. Molecular mechanisms involved in muscle wasting in cancer and ageing: cachexia versus sarcopenia. Int. J. Biochem. Cell Biol. 37, 1084–1104 (2005). 15743680 10.1016/j.biocel.2004.10.003

[b10] Tsuchida K. Activins, myostatin and related TGF-beta family members as novel therapeutic targets for endocrine, metabolic and immune disorders. Curr. Drug Targets: Immune, Endocr. Metab. Disord. 4, 157–166 (2004). 10.2174/1568008043339901 15180456

[b11] Lee S. -J. & McPherron A. C. Regulation of myostatin activity and muscle growth. Proc. Natl. Acad. Sci. USA 98, 9306–9311 (2001). 11459935 10.1073/pnas.151270098 PMC55416

[b12] McPherron A. C. & Lee S.-J. Double muscling in cattle due to mutations in the myostatin gene. Proc. Natl. Acad. Sci. USA 94, 12457–12461 (1997). 9356471 10.1073/pnas.94.23.12457 PMC24998

[b13] Clop A. *et al.* A mutation creating a potential illegitimate microRNA target site in the myostatin gene affects muscularity in sheep. Nat. Genet. 38, 813– 818 (2006). 16751773 10.1038/ng1810

[b14] Schuelke M. *et al.* Myostatin mutation associated with gross muscle hypertrophy in a child. New Engl. J. Med. 350, 2682–2688 (2004). 15215484 10.1056/NEJMoa040933

[b15] Shi Y. & Massague J. Mechanisms of TGF-beta signaling from cell membrane to the nucleus. Cell 113, 685–700 (2003). 12809600 10.1016/s0092-8674(03)00432-x

[b16] Trendelenburg A. U. *et al.* Myostatin reduces Akt/TORC1/p70S6K signaling, inhibiting myoblast differentiation and myotube size. Am. J. Physiol. Cell Physiol. 296, C1258–1270 (2009). 19357233 10.1152/ajpcell.00105.2009

[b17] Bodine S. C. *et al.* Akt/mTOR pathway is a crucial regulator of skeletal muscle hypertrophy and can prevent muscle atrophy *in vivo*. Nat. Cell Biol. 3, 1014–1019 (2001). 11715023 10.1038/ncb1101-1014

[b18] Pallafacchina G., Calabria E., Serrano A. L., Kalhovde J. M. & Schiaffino, S. A protein kinase B-dependent and rapamycin-sensitive pathway controls skeletal muscle growth but not fiber type specification. Proc. Natl. Acad. Sci. USA 99, 9213–9218 (2002). 12084817 10.1073/pnas.142166599 PMC123120

[b19] Zhao J. *et al.* FoxO3 coordinately activates protein degradation by the autophagic/lysosomal and proteasomal pathways in atrophying muscle cells. Cell Metabolism 6, 472–483 (2007). 18054316 10.1016/j.cmet.2007.11.004

[b20] Stitt T. N. *et al.* The IGF-1/PI3K/Akt pathway prevents expression of muscle atrophy-induced ubiquitin ligases by inhibiting FoXO transcription factors. Mol. Cell 14, 395–403 (2004). 15125842 10.1016/s1097-2765(04)00211-4

[b21] Leger B. *et al.* Akt signalling through GSK-3beta, mTOR and Foxo1 is involved in human skeletal muscle hypertrophy and atrophy. J. Physiol. 576, 923–933 (2006). 16916907 10.1113/jphysiol.2006.116715 PMC1890416

[b22] Mammucari C. *et al.* FoxO3 controls autophagy in skeletal muscle *in vivo*. Cell Metabolism 6, 458–471 (2007). 18054315 10.1016/j.cmet.2007.11.001

[b23] Mammucari C., Schiaffino S. & Sandri M. Downstream of Akt: FoxO3 and mTOR in the regulation of autophagy in skeletal muscle. Autophagy 4, 524–526 (2008). 18367868 10.4161/auto.5905

[b24] Zhao W. *et al.* Dependence of dexamethasone-induced Akt/FOXO1 signaling, upregulation of MAFbx, and protein catabolism upon the glucocorticoid receptor. Biochem. Biophys. Res. Commun. 378, 668–672 (2009). 19059383 10.1016/j.bbrc.2008.11.123

[b25] Han H. Q., Zhou X., Mitch W. E. & Goldberg A. L. Myostatin/activin pathway antagonism: molecular basis and therapeutic potential. Int. J. Biochem. Cell Biol. 45, 2333–2347 (2013). 23721881 10.1016/j.biocel.2013.05.019

[b26] McPherron A. C., Lawler A. M. & Lee S. J. Regulation of skeletal muscle mass in mice by a new TGF-beta superfamily member. Nature 387, 83–90 (1997). 9139826 10.1038/387083a0

[b27] Nakatani M. *et al.* Transgenic expression of a myostatin inhibitor derived from follistatin increases skeletal muscle mass and ameliorates dystrophic pathology in mdx mice. FASEB J. 22, 477–487 (2008). 17893249 10.1096/fj.07-8673com

[b28] Benabdallah B. F. *et al.* Inhibiting myostatin with follistatin improves the success of myoblast transplantation in dystrophic mice. Cell Transplant. 17, 337–350 (2008). 18522236 10.3727/096368908784153913

[b29] Winbanks C. E. *et al.* Follistatin-mediated skeletal muscle hypertrophy is regulated by Smad3 and mTOR independently of myostatin. J. Cell Biol. 197, 997–1008 (2012). 22711699 10.1083/jcb.201109091 PMC3384410

[b30] Kota J. *et al.* Follistatin gene delivery enhances muscle growth and strength in nonhuman primates. Sci. Transl. Med. 1, 6ra15 (2009). 10.1126/scitranslmed.3000112 PMC2852878 20368179

[b31] Chen J. L. *et al.* Elevated expression of activins promotes muscle wasting and cachexia. FASEB J. 28, 1711–1723 (2014). 24378873 10.1096/fj.13-245894

[b32] Rose F. F. Jr., Mattis V. B., Rindt H. & Lorson C. L. Delivery of recombinant follistatin lessens disease severity in a mouse model of spinal muscular atrophy. Hum. Mol. Genet. 18, 997–1005 (2009). 19074460 10.1093/hmg/ddn426 PMC2649020

[b33] Mendell J. R. *et al.* A phase 1/2a follistatin gene therapy trial for becker muscular dystrophy. Mol. Ther. 23, 192–201 (2015). 25322757 10.1038/mt.2014.200 PMC4426808

[b34] Herbison G. J., Jaweed M. M. & Ditunno J. F. Muscle atrophy in rats following denervation, casting, inflammation, and tenotomy. Arch. Phys. Med. Rehabil. 60, 401–404 (1979). 496606

[b35] Sacheck J. M. *et al.* Rapid disuse and denervation atrophy involve transcriptional changes similar to those of muscle wasting during systemic diseases. FASEB J. 21, 140–155 (2007). 17116744 10.1096/fj.06-6604com

[b36] Martyn J. A., Fagerlund M. J. & Eriksson L. I. Basic principles of neuromuscular transmission. Anaesthesia 64, 1–9 (2009). 19222426 10.1111/j.1365-2044.2008.05865.x

[b37] Zhou H. *et al.* Transgenic rat model of neurodegeneration caused by mutation in the TDP gene. PLoS Genet. 6, e1000887 (2010). 20361056 10.1371/journal.pgen.1000887 PMC2845661

[b38] Allen J. A., Stein R., Baker R. A. & Royden Jones H. Muscle atrophy associated with multiple sclerosis: A benign condition or the onset of amyotrophic lateral sclerosis? J. Clin. Neurosci. 15, 706–708 (2008). 18395451 10.1016/j.jocn.2007.04.024

[b39] Schoser B. G. H., Wehling S. & Blottner D. Cell death and apoptosis-related proteins in muscle biopsies of sporadic amyotrophic lateral sclerosis and polyneuropathy. Muscle Nerve 24, 1083–1089 (2001). 11439385 10.1002/mus.1114

[b40] Bialek P. *et al.* Distinct protein degradation profiles are induced by different disuse models of skeletal muscle atrophy. Physiol. Genomics 43, 1075–1086 (2011). 21791639 10.1152/physiolgenomics.00247.2010 PMC3217324

[b41] Agbulut O. *et al.* Slow myosin heavy chain expression in the absence of muscle activity. Am. J. Physiol. Cell Physiol. 296, C205–214 (2009). 18945940 10.1152/ajpcell.00408.2008

[b42] Haidet A. M. *et al.* Long-term enhancement of skeletal muscle mass and strength by single gene administration of myostatin inhibitors. Proc. Natl. Acad. Sci. USA 105, 4318–4322 (2008). 18334646 10.1073/pnas.0709144105 PMC2393740

[b43] Nakatani M. *et al.* Transgenic expression of a myostatin inhibitor derived from follistatin increases skeletal muscle mass and ameliorates dystrophic pathology in mdx mice. FASEB J. 22, 477–487 (2008). 17893249 10.1096/fj.07-8673com

[b44] Greenhaff P. L. *et al.* Disassociation between the effects of amino acids and insulin on signaling, ubiquitin ligases, and protein turnover in human muscle. Am. J. Physiol. Endocrinol. Metabol. 295, E595–604 (2008). 10.1152/ajpendo.90411.2008 PMC2536736 18577697

[b45] Raffaello A. *et al.* JunB transcription factor maintains skeletal muscle mass and promotes hypertrophy. J. Cell Biol. 191, 101–113 (2010). 20921137 10.1083/jcb.201001136 PMC2953439

[b46] Quy P. N., Kuma A., Pierre P. & Mizushima N. Proteasome-dependent activation of mammalian target of rapamycin complex 1 (mTORC1) is essential for autophagy suppression and muscle remodeling following denervation. J. Biol. Chem. 288, 1125–1134 (2013). 23209294 10.1074/jbc.M112.399949 PMC3542997

[b47] MacDonald E. M. *et al.* Denervation atrophy is independent from Akt and mTOR activation and is not rescued by myostatin inhibition. Dis. Model. Mech. 7, 471–481 (2014). 24504412 10.1242/dmm.014126 PMC3974457

[b48] Tang H. *et al.* mTORC1 promotes denervation-induced muscle atrophy through a mechanism involving the activation of FoxO and E3 ubiquitin ligases. Science Signaling 7, ra18 (2014). 24570486 10.1126/scisignal.2004809

[b49] Chen J. L. *et al.* Development of novel activin-targeted therapeutics. Mol. Ther. 23, 434–444 (2015). 25399825 10.1038/mt.2014.221 PMC4351455

[b50] Zhang D., Liu M., Ding F. & Gu X. Expression of myostatin RNA transcript and protein in gastrocnemius muscle of rats after sciatic nerve resection. J. Muscle Res. Cell Motil. 27, 37–44 (2006). 16450055 10.1007/s10974-005-9050-5

[b51] Baumann A. P., Ibebunjo C., Grasser W. A. & Paralkar V. M. Myostatin expression in age and denervation-induced skeletal muscle atrophy. J. Musculoskelet. Neuronal Interact. 3, 8–16 (2003). 15758361

[b52] Sakuma K. *et al.* The adaptive response of transforming growth factor-beta 2 and -beta RII in the overloaded, regenerating and denervated muscles of rats. Acta Neuropathol. 99, 177–185 (2000). 10672325 10.1007/pl00007422

[b53] Ozawa J. *et al.* Regulation of connective tissue remodeling in the early phase of denervation in a rat skeletal muscle. Biomed. Res. 34, 251–258 (2013). 24190237 10.2220/biomedres.34.251

[b54] Zhou X. *et al.* Reversal of cancer cachexia and muscle wasting by ActRIIB antagonism leads to prolonged survival. Cell 142, 531–543 (2010). 20723755 10.1016/j.cell.2010.07.011

[b55] Miller T. M. *et al.* Gene transfer demonstrates that muscle is not a primary target for non-cell-autonomous toxicity in familial amyotrophic lateral sclerosis. Proc. Natl. Acad. Sci. USA 103, 19546–19551 (2006). 17164329 10.1073/pnas.0609411103 PMC1748262

[b56] Chai R. J., Vukovic J., Dunlop S., Grounds M. D. & Shavlakadze T. Striking denervation of neuromuscular junctions without lumbar motoneuron loss in geriatric mouse muscle. PLoS One 6, e28090 (2011). 22164231 10.1371/journal.pone.0028090 PMC3229526

[b57] Gregorevic P., Blankinship M. J., Allen J. M. & Chamberlain J. S. Systemic microdystrophin gene delivery improves skeletal muscle structure and function in old dystrophic mdx mice. Mol. Ther. 16, 657–664 (2008). 18334986 10.1038/mt.2008.28 PMC2650831

[b58] Gregorevic P. *et al.* Systemic delivery of genes to striated muscles using adeno-associated viral vectors. Nat. Med. 10, 828–834 (2004). 15273747 10.1038/nm1085 PMC1365046

[b59] Dyck D. J. *et al.* Functional differences in lipid metabolism in resting skeletal muscle of various fiber types. Am. J. Physiol. Endocrinol. Metabol. 272, E340–E351 (1997). 10.1152/ajpendo.1997.272.3.E340 9124537

[b60] Clark A. S. & Mitch W. E. Comparison of protein synthesis and degradation in incubated and perfused muscle. Biochem. J. 212, 649–653 (1983). 6349623 10.1042/bj2120649 PMC1153139

[b61] Goldspink D. F., Garlick P. J. & McNurlan M. A. Protein turnover measured *in vivo* and *in vitro* in muscles undergoing compensatory growth and subsequent denervation atrophy. Biochem. J. 210, 89–98 (1983). 6189483 10.1042/bj2100089 PMC1154193

[b62] Rasband W. S. ImageJ U.S. National Institutes of Health, Bethesda, Maryland, USA, http://imagej.nih.gov/ij/, (1997–2015).

